# From METS to malaria: RRx-001, a multi-faceted anticancer agent with activity in cerebral malaria

**DOI:** 10.1186/s12936-015-0720-5

**Published:** 2015-05-28

**Authors:** Ozlem Yalcin, Bryan Oronsky, Leonardo J. M. Carvalho, Frans A. Kuypers, Jan Scicinski, Pedro Cabrales

**Affiliations:** Department of Bioengineering, University of California, 9500 Gilman Dr., La Jolla, San Diego, CA 92093-0412 USA; School of Medicine, Koç University, Sariyer, Istanbul, Turkey; EpicentRx Inc, Mountain View, CA USA; Center for Malaria Research, La Jolla Bioengineering Institute, San Diego, CA USA; Laboratory of Malaria Research, Oswaldo Cruz Institute, Fiocruz, Rio de Janeiro Brazil; Children’s Hospital Oakland Research Institute, Oakland, CA USA

**Keywords:** Cerebral malaria, Pial microcirculation, Anemia, G6PD, Nitric oxide, Epigenetic, RRx-001

## Abstract

**Background:**

The survival of malaria parasites, under substantial haem-induced oxidative stress in the red blood cells (RBCs) is dependent on the pentose phosphate pathway (PPP). The PPP is the only source of NADPH in the RBC, essential for the production of reduced glutathione (GSH) and for protection from oxidative stress. Glucose-6-phosphate dehydrogenase (G6PD) deficiency, therefore, increases the vulnerability of erythrocytes to oxidative stress. In *Plasmodium*, G6PD is combined with the second enzyme of the PPP to create a unique bifunctional enzyme, named glucose-6-phosphate dehydrogenase–6-phosphogluconolactonase (G6PD-6PGL). RRx-001 is a novel, systemically non-toxic, epigenetic anticancer agent currently in Phase 2 clinical development for multiple tumour types, with activity mediated through increased nitric oxide (NO) production and PPP inhibition. The inhibition of G6PD and NO overproduction induced by RRx-001 suggested its application in cerebral malaria (CM).

**Methods:**

*Plasmodium berghei* ANKA (PbA) infection in C57BL/6 mice is an experimental model of cerebral malaria (ECM) with several similar pathological features to human CM. This study uses intravital microscopy methods with a closed cranial window model to quantify cerebral haemodynamic changes and leukocyte adhesion to endothelial cells in ECM.

**Results:**

RRx-001 had both single agent anti-parasitic activity and significantly increased the efficacy of artemether. In addition, RRx-001 preserved cerebral perfusion and reduced inflammation alone or combined with artemether. RRx-001’s effects were associated with inhibition of PPP (G6PD and G6PD-6PGL) and by improvements in microcirculatory flow, which may be related to the NO donating properties of RRx-001.

**Conclusion:**

The results indicate that RRx-001 could be used to potentiate the anti-malarial action of artemisinin, particularly on resistant strains, and to prevent infection.

## Background

Cerebral malaria (CM) is the most severe neurological complication of malarial infection. CM is a leading cause of neurodisability and death [1]. As a multisystem and multiorgan disease, a range of clinical symptoms characterizes CM, they include unarousable coma, encephalopathy, seizures, severe anemia and metabolic acidosis [1–3]. Its pathogenesis is quite complex and involves a cascading interaction between the parasitized red blood cells (RBCs), and cytokines and endothelial receptors, leading to reduced regional, or even global, cerebral blood flow with stagnation hypoxia and neurologic sequelae, inflammatory responses, breakdown of the blood brain barrier and cerebral microhaemorrhages [1, 4, 5].

The malarial parasitic protozoan, *Plasmodium falciparum*, inextricably linked with humanity since the dawn of time and the evolutionary driving force behind sickle cell disease, thalassaemias and G6PD deficiency, and responsible for more than a million deaths every year, remains an elusive therapeutic target [6]. The high mortality rate is partly due to the lateness of presentation i.e. after the development of loss of consciousness or coma, and is a reflection of rapid disease progression, limited access to rapid diagnosis and treatment. An increasing prevalence of resistance to anti-malarial drugs and a relative dearth of active agents compound the problem: adjuvant therapy clinical trials with mannitol, steroids, antipyretics, anti-seizure control, pentoxifylline, and deferoxamine, among others, have yielded disappointing results [7].

Cellular redox reactions play important roles not only in redox regulatory processes and antioxidant defense, but also in the mechanisms of drug action and drug resistance in malaria parasites. Recent advances in parasite biology suggest a compartmentalization of redox metabolism and cellular processes that are sites of drug action [8, 9]. The pentose phosphate pathway (PPP) of both the host RBCs and the parasite are crucial to neutralize deleterious reactive oxygen species (ROS) that accumulate during RBC parasite infection and from haemoglobin (Hb) degradation [10]. Since RBCs lack mitochondria, the PPP is the only pathway in these cells to generate NADPH, which acts as the main reducing equivalent, contributing to the maintenance of reduced glutathione (GSH) [11]. As a result, the PPP of erythrocytes is highly up-regulated after Plasmodium infection, due to high oxidative stress requiring NADPH redox power for antioxidant systems, and the need for ribose 5-phosphate to synthesize nucleotides for cell growth [12]. On the other hand, the first two steps of the PPP in the parasites (*P. falciparum, Plasmodium berghei, Plasmodium knowlesi, Plasmodium yoelii* and *Plasmodium chabaudi*) are catalyzed by a bifunctional enzyme, glucose-6-phosphate dehydrogenase-6-phosphogluconolactonase (G6PD-6PGL) [13]. While a Krebs cycle is present and functional in the malaria parasite, glucose flux through it is reduced and depends on the developmental stage of the parasite [14]. By contrast, glucose utilization through glycolysis and PPP increases ~70-fold with *P. falciparum* infection and, remarkably, the enhancement in activity is primarily due to activation of the parasite PPP [15].

C57BL/6 mice infected with *P. berghei*, strain ANKA (PbA), develop neurological symptoms (ataxia, progressive paralysis, hypothermia, seizure, coma) characteristic of a syndrome known as experimental cerebral malaria (ECM) [16]. Intravital microscopy, which allows direct observation of the live brain in real time through a closed cranial window model, is unsurpassed in terms of the ability to explore the cerebral microvasculature during disease progression [17–20]. The cortical microcirculation shares the same general behaviour and functionality of the brain circulation, including the blood—brain barrier properties [21]. ECM pathogenesis is associated with microcirculatory complications, similar to that observed in parenchymal vessels, including decreased blood flow, vasoconstriction, and vascular plugging by adherent cells and microhaemorrhages [18–20].

RRx-001 is a redox-active epigenetic anticancer agent in Phase 2 clinical trials with p53 and Nrf2-mediated neuroprotective properties (unpublished data P. Cabrales) and potential anti-sickling capabilities [22]. RRx-001 selectively binds to the β-cysteine 93 residue on Hb [23]. Although Hb is not technically an enzyme, it functions as a nitrite (NO_2_^−^) reductase, reducing nitrite into nitric oxide (NO) in the presence of hypoxia [24]. Binding of RRx-001 to the β-cysteine 93 residue allosterically increases inherent Hb nitrite reductase functionality to overproduce NO, specifically under hypoxic conditions [25].

The present study defines the efficacy of RRx-001, artemether, and a combination of artemether-RRx-001 in a murine model of *P. berghei* ECM as determined by (i) *P. berghei* parasitaemia kinetics with treatment starting on day 7 post infection (ii) survival of mice and (iii) motor performance of mice with late-stage ECM. In addition, this study evaluates the *in vitro* effect of RRx-001 on G6PD activity, the *in vitro* anti-malarial activity of RRx-001, and its limited haemolytic effects.

## Methods

### Blood collection

Blood collection was approved by the Institutional Animal Care and Use Committee, and was conducted accordingly to the Guide for the Care and Use of Laboratory Animals (US National Research Council, 2010). Blood was obtained from donor mice (C57BL/6, ~25 g). Animals were anaesthetized (pentobarbital 60 mg/kg ip) and a femoral catheter (PE-50) was implanted and blood was drawn into syringes containing ACD (38 mM citric acid, 75 mM sodium citrate, 136 mM glucose) as the anticoagulant. The cells were pelleted, buffy coat was discarded to remove the leukocytes, and the erythrocytes were washed three times (RPMI 1640 supplemented with 27 mM NaHCO_3_, 25 mM HEPES, 0.35 mM hypoxanthine). The washed RBCs were then resuspended in RPMI 1640 with 0.5 % albumin solution. Asexual stages of *P. berghei* were cultured *in vitro* and synchronized by sorbitol [26]. Briefly, the cells were harvested when maximum infected RBCs (iRBCs) were predominantly rings, washed and treated with 5 % sorbitol (in double distilled water) at 37 °C for 10 min, washed repeatedly with RPMI 1640, and subcultured with RBCs prepared as described above. Parasites were maintained at 5 % haematocrit at 37 °C in a humidified chamber containing 5 % CO_2_.

### *In vitro* glucose consumption

IRBCs were harvested, washed and resuspended at 50 % haematocrit in RPMI 1640. Glucose consumption was determined by incubating 1 mL aliquots of IRBCs (trophozoite stage) and uninfected RBCs at 37 °C. Glucose concentration in those aliquots was increased by adding glucose solution to 12 mM. Samples (100 μL) were taken immediately before and at 30, 60, 120, 180, and 240 min after adding glucose, and plasma separated by centrifuging at 10,000 g for 2 min. Glucose concentration was determined using a YSI 2300 STAT Plus (YSI, Yellow Springs, Ohio) and glucose consumption was calculated from a linear regression of glucose concentration versus time. For glucose consumption of free parasites, the IRBCs (trophozoite stage) were treated with Sendai virus *(*Sigma*-*Aldrich*).* Briefly, iRBCs (5 % haematocrit) were incubated with Sendai virions (40 μg/mL) for 7 min. IRBC, uninfected RBCs and free trophozoite parasites were also evaluated in medium containing 0.5 mM methylene blue (MB).

### Closed cranial window animal preparation

Animal handling and care followed the NIH Guide for Care and Use of Laboratory Animals. All protocols were approved by the Institutional Animal Care and Use Committee, and conducted accordingly to the Guide for the Care and Use of Laboratory Animals (US National Research Council, 2010). Eight to 10-week old C57Bl/6 (Jackson Laboratories, ME) were implanted with a closed cranial window model as described elsewhere [27]. Briefly, mice were anesthetized with ketamine-xylazine and were administered dexamethasone (0.2 mg/kg), carprofen (5 mg/Kg) and ampicillin (6 mg/kg) subcutaneously, in order to prevent post-surgical swelling of the brain, inflammatory response and infection. After shaving the head and cleansing with ethanol 70 % and betadine, the mouse was placed on a stereotaxic frame and the head immobilized using ear bars. The scalp was removed with sterilized surgical instruments; lidocaine-epinephrine was applied on the periosteum, which was then retracted to expose the skull. A 3–4 mm diameter skull opening was made in the left parietal bone using a surgical drill. Under a drop of saline, the craniotomy was lifted away from the skull with very thin tip forceps and gelfoam previously soaked in saline applied to the dura mater in order to stop any eventual small bleeding. The exposed area was covered with a 5 mm glass cover slip secured with cyanocrylate-based glue and dental acrylic. Carprofen and ampicillin were given daily for 3–5 days after recovery from surgery. Mice presenting signs of pain or discomfort were euthanized with 100 mg/kg of euthasol IP. Two-three weeks after surgery, mice fulfilling the inclusion criteria (see below) were inoculated with *P. berghei* ANKA and, on day 6 of infection, they were lightly anesthetized with isoflurane (4 % for induction, 1–2 % for maintenance) and held on a stereotaxic frame for measurements of pH and PO_2_.

### Inclusion criteria

Animals were suitable for the experiments if: 1) animal behaviour was normal and; 2) microscopic (x350 magnification) examination of the cranial window did not reveal signs of edema or bleeding.

### Parasite infection

Animals were inoculated with an IP injection of 1 × 10^6^*P. berghei* ANKA parasites expressing the green fluorescent protein (PbA-GFP, a donation from the Malaria Research and Reference Reagent Resource Center – MR4, Manassas, VA). Parasitaemia, body weight, rectal temperature and clinical status (using six simple tests adapted from the SHIRPA protocol, as previously described) were monitored daily from day 4 of the infection [28]. Parasitaemia was checked using flow cytometry by detecting the number of fluorescent GFP-expressing pRBCs in relation to 10,000 RBCs. ECM was diagnosed when one or more of the following clinical signs of neurological involvement were observed: ataxia, limb paralysis, poor righting reflex, seizures, roll-over or coma.

### Experimental groups

At day 7, *P. berghei*-infected mice presenting poor righting reflex, hypothermia and/or other clinical signs of neurological involvement such as ataxia, limb paralysis, seizures and/or roll-over were randomly assigned to the four different treatment therapies. Group 1: Untreated animals. Group 2: Animals were treated with the experimental agent, RRx-001 (EpicentRx, Inc Mountain View, CA) IV at 10 mg/kg. Group 3: Animals were treated with artemether (Artesiane, Dafra Pharma, Belgium) IP at 50 mg/kg. Group 4: Animals were treated with artemether IP at 50 mg/kg and RRx-001 IV at 10 mg/kg. Treatments were given every other day starting on day 7 after infection until day 12 after infection. Parasitaemia, motor behaviour and rectal temperature were checked at each time point and daily afterwards. After treatment, parasitaemia was checked by microscopical examination of Giemsa-stained blood smears to differentiate viable from dead parasites.

### Experimental set-up

Animals were lightly anesthetized with isoflurane (4 % for induction, 1–2 % for maintenance). They were secured to the microscopic stage of an intravital microscope (BX51WI, Olympus, New Hyde Park, NY) on a stereotaxic frame with the head gently held with ear bars for epi-illumination imaging. Body temperature, measured pre-anesthesia, was maintained with a heating pad. The tissue image was projected onto a charge-coupled device camera (COHU 4815) connected to a videocassette recorder and viewed on a monitor. Measurements were carried out using a 40X (LUMPFL-WIR, numerical aperture 0.8, Olympus) water immersion objective. The animals did not recover from anesthesia, being euthanized (Euthasol 100 mg/kg, IP) right after the intra vital microscopy measurements.

### Microhaemodynamics

A video image-shearing method was used to measure vessel diameter (D) [29]. Changes in arteriolar and venular diameter from baseline were used as indicators of a change in vascular tone. Arteriolar and venular centerline velocities were measured on-line using the photodiode cross-correlation method (Photo Diode/Velocity Tracker Model 102B, Vista Electronics, San Diego, CA). The measured centerline velocity (V) was corrected according to vessel size to obtain the mean RBC velocity [30]. Blood flow (Q) was calculated from the measured values as Q = π × V (D/2)^2^. This calculation assumes a parabolic velocity profile and has been found to be applicable to tubes of 15–80 μm internal diameters and for Hcts in the range of 6–60 % [30].

### Endothelial leukocyte adhesion

The closed cranial window model was used as previously described [19]. On day 8 after *P. berghei* infection, anti-CD45-TxR antibodies (CalTag, Carlsbad, CA; 4 μg) were intravenously infused through the tail vein (volume: 50 μL). Using water-immersion objectives (20X), blood vessel images were captured using a CCD camera (COHU 4815, San Diego, CA). Fifteen minutes after injection red (615 nm) fluorescently labeled leukocytes were excited and images were captured with a Vivid Standard: XF42 filter. Adherence was defined as cells remaining static for 30 s. For each selected vessel, Added leukocytes were quantified in a 100 μm length sections.

### Haematocrit and haemoglobin

Blood was collected from the tail in heparinized glass capillaries. Haemoglobin was determined spectrophotometrically from a single drop of blood B-haemoglobin (Hemocue, Stockholm, Sweden). Haematocrit was estimated by centrifugation.

### *In vitro* drug sensitivity testing

A series of experiments were conducted to evaluate the anti-malarial activity of RRx-001 on blood stage malaria parasites using *P. berghei* in mice RBCs. Infected RBCs from a continuous *in vitro* culture of asexual erythrocyte stages of *P. berghei* were used for the anti-malarial assay. [31]. Different doses of RRx-001 and positive controls, chloroquine diphosphate (Sigma-Aldrich, St. Louis, MO) and artemether (Artesiane®, Dafra Pharma, Belgium), were incubated with the parasites for 72 h followed by detection of growth inhibition using the DNA binding dye SYBR green [32, 33]. Briefly, 20 μL of 10X SYBR Green I nucleic acid gel stain (Sigma-Aldrich), 0.5 % v/v Triton, and 0.5 mg/mL saponin solution were added per 80 uL of treated infected RBCs per well. Assay plates were shaken for 30 s, incubated in the dark for 4 h, and then read with on a using a microplate reader (Synergy HT, Bio-Tek Instruments, Inc., Winooski, VT) at Ex/Em 485 nm/535 nm. EC50s were calculated using GraphPad Prism 6 (GraphPad Software, Inc., San Diego, CA).

### G6PD activity

Cell free extracts were used for measuring G6PD activity. Dehydroepiandrosterone (DHEA, Sigma-Aldrich) a potent uncompetitive inhibitor of mammalian G6PD was used as positive control. HEP-G2 cells (hepatocellular carcinoma, ATCC, Manassas, VA) were seeded and when the cells were about 60–70 % confluent, the medium from each flask was discarded, and the attached cells were rinsed twice with phosphate buffered saline (PBS). Cells were then harvested and centrifuged in PBS. Aliquots were used for determination of protein concentration, following bicinchoninic acid (BCA) protein assay (Pierce Biotech. Inc). Cell extract was incubated in a final volume of 150 μL of 100 mM potassium phosphate buffer (pH 7.4) containing a NADPH generating system. The reaction was started by the addition of 20 μL of cell extracts to 980 μL of 0.1 M Tris–HCl buffer (pH 8.1) containing 1 M MgCl_2_,10 mM NADP, 200 mM glucose-6-phosphate, and testing compounds (RRx-001 and DHEA). These mixtures were incubated at room temperature. Rate of NADPH formation was determined using the absorption at 340 nm (Lambda 20, Perkin-Elmer, Foster City, CA). The result was expressed as a percentage of the control activity measured with the cell extract incubated in the presence of DMSO.

### Haemolytic activity in uninfected mice

Nine (*n* = 9) uninfected mice were treated with RRx-001 (EpicentRx, Inc.) IV at 10 mg/kg (*n* = 3), artemether (Dafra Pharma) IP at 50 mg/kg (*n* = 3), or artemether IP at 50 mg/kg and RRx-001 IV at 10 mg/kg (*n* = 3). Blood was collected before treatment, 24 h and 48 h after treatment from the tail in heparinized glass capillaries.

### Data analysis

Results are presented as mean ± standard deviation, except for vessel diameters and blood flows. Data between groups was analysed by an analysis of variance (ANOVA, Kruskal-Wallis test). When appropriate, *post hoc* analyses were performed with the Dunns multiple comparison test. All statistics were calculated using GraphPad Prism 6 (GraphPad Software, Inc., San Diego, CA). Changes were considered statistically significant if *P* < 0.05.

## Results

### RRx-001 is a potent inhibitor of G6PD

**Fig. 1:**
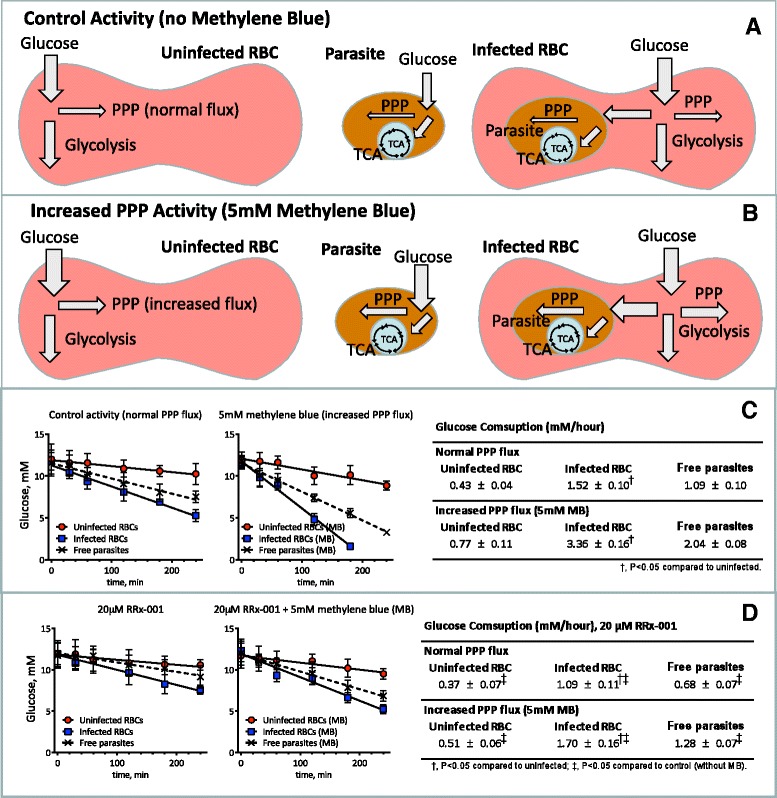
**a**. Illustration of glycolytic metabolic fluxes during normal conditions. Previous studies indicate that the PPP uses 30 % of the glucose consumed by the RBC [43]. **b**. Illustration of glycolytic metabolic fluxes during increased PPP flux resulting from methylene blue. The response of the RBC PPP to methylene blue has been widely used to demonstrate the presence of PPP in RBC [44]. **c**. Glucose consumption of Plasmodium berghei ANKA (PbA) infected RBCs, uninfected RBCs and free parasites. Parasitaemia was 10 % of the infected RBC. Glucose utilization of infected RBCs was 35 times higher than uninfected RBCs. Free parasites accounted for the increased in glucose consumption. Glucose consumption during increased PPP flux with methylene blue (MB, 5 mM) augmented glucose utilization of uninfected RBCs by 80 % compared to control conditions. Infected RBCs increased PPP flux in response to MB by more than 120 %, and the free parasite increased PPP flux in response to MB by 100 %. Therefore, the parasite and infected RBCs have large capacity to deal with oxidative stress, and the uninfected RBCs to preserve reduced Hb and GSH. **d**. Glucose consumption of *Plasmodium berghei* ANKA infected RBCs, uninfected RBCs and free parasites treated with 20 μM RRx-001. RRx-001 reduced glucose utilization of uninfected, infected RBCs and parasites. RRx-001 also prevented the drastic increase in glucose consumption during of increased PPP flux with MB (5 mM). Although, MB still increased the glucose utilization of RRx-001 treated uninfected RBCs by 35 %, in the infected RBCs by 55 %, and in the free parasite by 80 %, respectively. Therefore, RRx-001 reduced the capacity of parasites, infected RBCs and uninfected RBCs to deal with oxidative stress, and to preserve reduced Hb and GSH

Given the tropism of RRx-001 for the RBC and the dependence of the malaria parasite on the PPP, the effects of RRx-001 on 6GPD of RBCs, infected RBCs, and parasites were initially explored (Fig. 1c). Glucose consumption was lower in RRx-001 (20 μM) treated RBC infected compared to untreated infected cells, indicating inhibition of the PPP (Fig. 1d). 5 mM methylene blue, which stimulates PPP flux, did not increase glucose utilization of treated cells in combination with RRx-001 (Fig. 1c and 1d). Infected RBCs presented a significantly increased glucose metabolism compared to uninfected RBCs (Fig. 1). Both glycolysis and PPP appear to be increased in the infected RBCs. Similarly, previous studies have shown that the ratio of glucose metabolization through the PPP and glycolysis increases for infected relative to uninfected RBCs [15]. The glucose utilization by the infected RBCs in the animals is relatively small compared to the overall glucose utilization by the other tissues. However, previous studies have investigated glucose uptake rates into brain tissue in *P. berghei*-infected mice, and found there to be no significant difference in uptake rates compared with uninfected mice [34].

### Adjunctive therapy with RRx-001 improves survival in mice with late-stage ECM

**Fig. 2:**
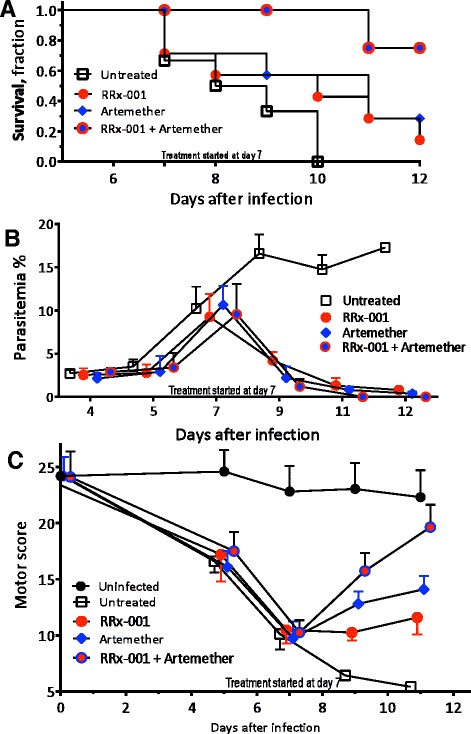
Survival, parasitaemia, and motor score of mice infected with Plasmodium berghei ANKA. Mice were either left untreated (*n* = 7; *white quare*) or were treated with RRx-001 (IV at 10 mg/kg; *n* = 7 mice; *red circle*) or artemether (IP at 50 mg/kg; *n* = 7, *blue diamond*), or 10 mg/kg IV of RRx-001 plus 50 mg/kg IP of artemether (*n* = 7, *red circle* with *blue border*). All parameters were measured at the same time points for all groups. The points were artificially displaced horizontally for clarity. **a**. Mice survival after infection. These data represent the trajectory of disease. At day 7 post infection, Kaplan-Meier survival curves were plotted and by day 12 differences in rates of survival in the various groups were observed: RRx-001 plus artemether 83 %, RRx-001 16.6 %, artemether 50 % and untreated 0 %. **b**. Mice parasitaemia after infection. Parasitaemia was monitored daily in surviving mice with Giemsa-stained thin blood smears from day 1 to day 12 post infection, and the mean ± SD for each group is shown. The parasitaemia peaks for all groups occurred between days 7–9. The parasitaemia in the untreated group increased progressively from the peak, while RRx-001, artemether decreased to almost zero levels and RRx-001 + artemether reached zero by Day 11. The percent change in parasitaemia after initiation of treatment with the combination of artemether-RRx-001 reaches 100 %; while artemether alone and RRx-001 approaches did not reach 100 %. **c**. Neurological motor scores after infection. Motor performance (modified SHIRPA test) for all groups was analysed. Motor behaviour drastically deteriorated in the untreated group while it rebounded in the treated groups, only returning to baseline i.e. to the level of uninfected groups in the RRx-001 + artemether cohort

Starting on day 7 post-infection, every other day treatment resulted in 83 % survival in the RRx-001 plus artemether-treated group compared to a 50 % survival in the group receiving artemether alone. All untreated mice succumbed to their infection (Fig. 2a). In addition near zero parasitaemia was observed by Day 11, 4 days after the start of treatment with the combination of artemether-RRx-001 while parasitaemia in mice treated with RRx-001 and artemether monotherapy trended towards, but did not quite reach, zero parasitaemia by Day 12 (Fig. 2b). In the parasite infected untreated group hyperparasitaemia increased until death.

### RRx-001 adjunctive therapy rescues mice with established clinical signs of motor impairment

Mice infected with PbA exhibit similar neurological impairments to those in *P. falciparum* infected humans, including ataxia, hemiplegias, and seizures (Fig. 2c). As in humans, survival with ECM does not necessarily correlate with an improvement in neurological impairment. Therefore, mice were evaluated for motor performance, starting on Day 7 after infection, with five tests (transfer arousal, locomotor activity, tail elevation, wire maneuver and righting reflex) modified from the SHIRPA protocol [28]. For each test, mice received an individual score, and the sum of scores was used to create a composite score. The results **(**Fig. 2c) demonstrated that by Day 11, mice treated with the every other day combination of RRx-001 and artemether, signs of neurological involvement were rapidly responsive and returned to baseline while the mice treated with artemether alone and RRx-001 alone improved only slightly and the untreated cohort not at all. These data suggest that the combination of artemether and RRx-001 is neuroprotective and ameliorates neurological deficits in murine cerebral malaria.

### RRx-001 adjunctive treatment decreases inflammation and prevents severe anaemia

**Fig. 3:**
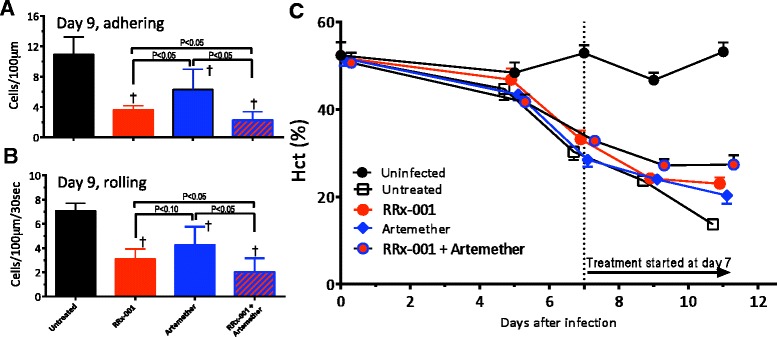
Beneficial effect of artemether plus RRx-001 treatment in leukocyte adhering and rolling in pial venules from late-stage ECM mice. Groups are displayed as follows: untreated *Plasmodium berghei* ANKA infected mice (*black*), PbA infected mice treated with RRx-001 (IV at 10 mg/kg, *red*), PbA infected mice treated with artemether (IP at 50 mg/kg *blue*) and PbA infected mice treated with 10 mg/kg IV of RRx-001 plus 50 mg/kg IP of artemether (*red* with *blue border*). **a**. Adhering leukocytes. Leukocyte adhesion in venules was measured (Day 9). **b**. Rolling leukocytes. Leukocyte rolling in venules was measured (Day 9). **c**. Haematocrit after infection. Mice were either left untreated (*n* = 7; *white quare*) or were treated with RRx-001 (IV at 10 mg/kg; *n* = 7 mice; *red circle*) or artemether alone (IP at 50 mg/kg; *n* = 7, *blue diamond*), or 10 mg/kg IV of RRx-001 plus 50 mg/kg IP of artemether (*n* = 7, *red circle* with *blue border*). Haematocrit was measured from samples collected from the tail vein. Hct were measured at the same time points for all groups. The points were artificially displaced horizontally for clarity

The number of adhered and rolling leukocytes in small venules on day 8 after infection were quantified using fluorescent intra-vital microscopy (Fig. 3a and 3b). All therapies reduced the number of adhered and rolling leukocytes compared to untreated mice. Treatment with RRx-001 and artemether + RRx-001 reduced the number of adhered and rolling leukocytes compared to artemether alone. All groups showed decrease haematocrit compared to uninfected mice (Fig. 3c). The RRx-001+ artemether combination prevented the decrease in haematocrit.

### RRx-001 adjunctive treatment improves cerebral haemodynamics

**Fig. 4:**
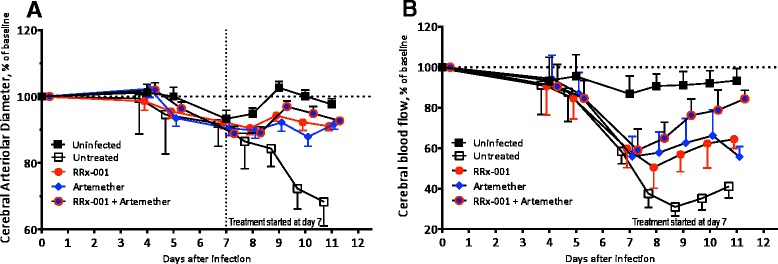
Beneficial effect of artemether plus RRx-001 treatment in pial arterioles and blood flow from late-stage ECM mice. Mice were either left untreated (*n* = 7; *white quare*) or were treated after day 7 with RRx-001 (IV at 10 mg/kg; *n* = 7 mice; *red circle*) or artemether alone (IP at 50 mg/kg; *n* = 7, *blue diamond*), or 10 mg/kg IV of RRx-001 plus 50 mg/kg IP of artemether (*n* = 7, *red circle* with *blue border*). **a**. Changes in arteriolar diameter relative to before infection. Vessel diameter remained unchanged until day 7. Untreated animals showed rapid progression of vasoconstriction. Treatment presented arteriolar vessel diameter compared to untreated. RRx-001 alone and in combination with artemether prevented the vasoconstriction. **b**. Changes in arteriolar blood flow relative to before infection. Blood flow decreased to 60 % of before infection by day 7 after infection. Blood flow in untreated animals continue decreasing to 20–30 % by day 9 after infection. Treatment presented blood flow at around 60 % before infection, with the exception that RRx-001 in combination with artemether restored blood flow to 80 % before infection

Using intra vital microscopy through a closed cranial window, pial arteriolar diameters and cerebral blood flow (RBC velocities) were evaluated in groups of seven animals. Non-infected control mice showed stable vessel diameters. All *P. berghei*-infected mice presented with vasoconstriction of pial arterioles compared with uninfected mice (Fig. 4a). Vessels in untreated mice remained constricted with a mean decrease in vascular patency of 40 %. By contrast, mice treated with artemether + RRx-001, artemether alone and RRx-001 alone showed reversal of vasoconstriction relative to the uninfected animals. All groups showed a mean 40 % decrease in blood flow on day 7 at the start of treatment (Fig. 4b). The RRx-001+ artemether combination attenuated the decrease in blood flow in relation to uninfected mice, returning almost to baseline levels, followed by artemether alone and RRx-001 alone where blood flow remained stable at around 60 % of baseline. Blood flow in the untreated *P. berghei*-infected mice dropped to around 40 % of baseline.

### RRx-001 anti-malarial activity, G6PD inhibition and haemolytic safety

*In vitro* drug susceptibility of infected mice RBC allowed to establish dose response for artemether and RRx-001 compared to chloroquine. *In vitro* drug susceptibility is presented in Table 1. RRx-001 showed potent anti-malarial, although as a single agent, the drug sensitivity testing indicated that higher dose of RRx-001 was required to inhibited 50 % of the parasite’s activity. RRx-001 effects on G6PD are presented in Table 2. In comparison to DHEA, a potent uncompetitive inhibitor of mammalian G6PD, RRx-001 showed a more potent inhibition at concentrations as low as 1 μM. Haemolytic potential of RRx-001 in uninfected animals was compared to artemether, and the combination RRx-001 and artemether at the dose used with the infected mice. Haemolytic potential of RRx-001 is presented in Table 3. All drugs and their combination showed mild haemolysis, most probably due to the G6PD inhibition and the increased oxidative stress.

**Table 1:** *In vitro* drug sensitivity testing

Antimalaria activity	
	IC50 (ug/ml)
RRx-001	0.14 ± 0.04
Artemether	0.09 ± 0.02
Chloroquine	0.05 ± 0.02

IC50 is the concentration inhibiting 50 % of parasite’s activity. Values are mean ± SD (*n* = 4)

**Table 2:** G6PD Activity

Effects on G6PD inhibition activity			
G6PD activity in HEP-G2 (% of control, 182 ± 16 nmol/min/mg of protien)			
	1 μM	50 μM	100 μM
DHEA	84 ± 7	39 ± 6	11 ± 2
RRx-001	78 ± 6	16 ± 4	6 ± 1

Cell-free extracts were incubated with each drug at concentrations of 1, 10 and 100 μM, and G6PD activities were measured. Dehydroepian drosterone (DHEA). Data represent the mean ± SD(*n* = 4)

**Table 3:** Hemolytic activity in uninfected mice

Hemolytic effects on RRx-001, Artemether, & RRx-001 + Artemether			
Hematocrit, %	BL	Day 1	Day 1
RRx-001	51 ± 2	49 ± 1	49 ± 2
Artemether	51 ± 2	47 ± 2	48 ± 2
RRx-001 + Arthemether	50 ± 2	46 ± 4	47 ± 2

Hematocrit was measured before (baseline, BL) and after single treatment of un infected mice. Three animals were used in each group. Values are reported as mean ± SD

## Discussion

Malaria is characterized by low NO production and endothelial dysfunction [35]. Conversely, malarial immunity is associated with high levels of NO [36, 37] and NO donation with nitroglycerin or L-arginine supplementation had been shown to partially prevent the development ECM, decrease vascular pathology, and increase survival when combined with artemether [37]. In addition, previous studies demonstrated the NO donors (such as NONOates, NO-generating diazeniumdiolate) can prevent ECM, however high doses are required [18, 20].

RRx-001 has a distinct advantages over nitroglycerin (and over other systemic vasodilators like nifedipine and NONOates) in the clinical setting because RRx-001 mediated NO delivery is site-specific under hypoxia; therefore, the orthostatic hypotension, tachycardia, headaches and palpitations associated with nitroglycerin and other nitrate esters or the severe hypotensive effects of NONOates do not occur with RRx-001. Safety data from preclinical studies showed blood pressure changes only at very high doses (EpicentRx, unpublished data) and it was, therefore, unnecessary to routinely measure blood pressure in the Phase I and II trials. In the Phase 1 study, RRx-001 was well-tolerated, systemically non-toxic and was not associated with changes in haemoglobin-associated variables [38]. The present study demonstrates that RRx-001 inhibits in a dose dependent fashion the PPP (G6PD) of RBCs and parasites. Analysis of PPP flux revealed that RRx-001 inhibits G6PD more effectively than the positive control, DHEA, a known G6PD inhibitor, preventing conversion of glucose-6-phosphate to 6-phosphogluconolactone and reduction of NADP. The significant G6PD inhibition observed with dose as low as 1 μM, could limits ribose phosphate, which is necessary for RNA and DNA biosynthesis in the parasite.

**Fig. 5:**
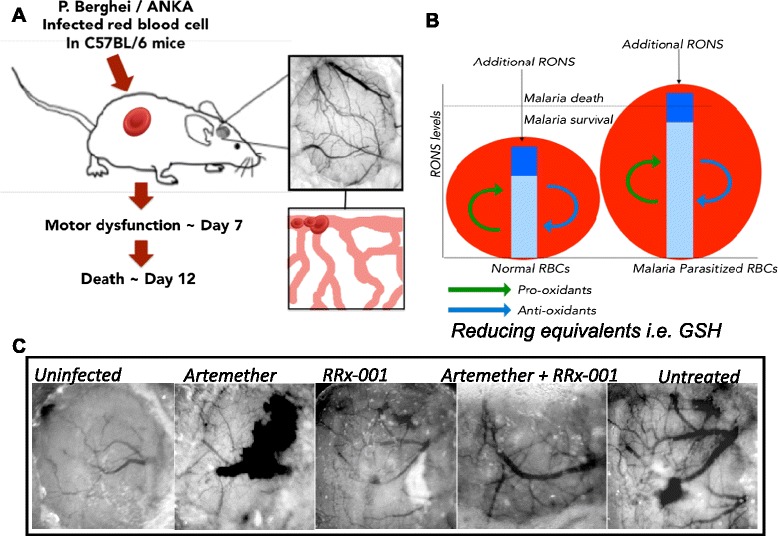
Graphical abstract of the mouse pial vascular network after infection with *P. Berghei* / ANKA. **a**. Progression of cerebral malarial in the rodent model. Neurological and motor dysfunction correlate with reduced cerebral blood flow and increased inflammation. **b**. Balance between pro-oxidant and anti-oxidant determines survival of erythrocytes. Limiting the capacity to deal with oxidative stress by inhibiting G6PD and limiting NADPH levels with RRx-001, increases efficacy of anti-malarial therapies. Intraerythrocytic malarial parasites are subject to high levels of free radicals due to the catabolism of Hb. Additional RONS stress from RRx-001 and artemether may tip the parasite over the threshold into death. **c**. Macroscopic images of cranial windows from the rodent model. Images are from day 9 after infection. Uninfected animals show normal microcirculation. Artemether treated animal showed micro-haemorrhages. RRx-001 treated animal showed preserved microcirculation structure. Artemether and RRx-001 treated animal showed preserved microcirculation structure and microvascular function. Untreated animals showed micro-haemorrhages followed by vasospasms

**Fig. 6:**
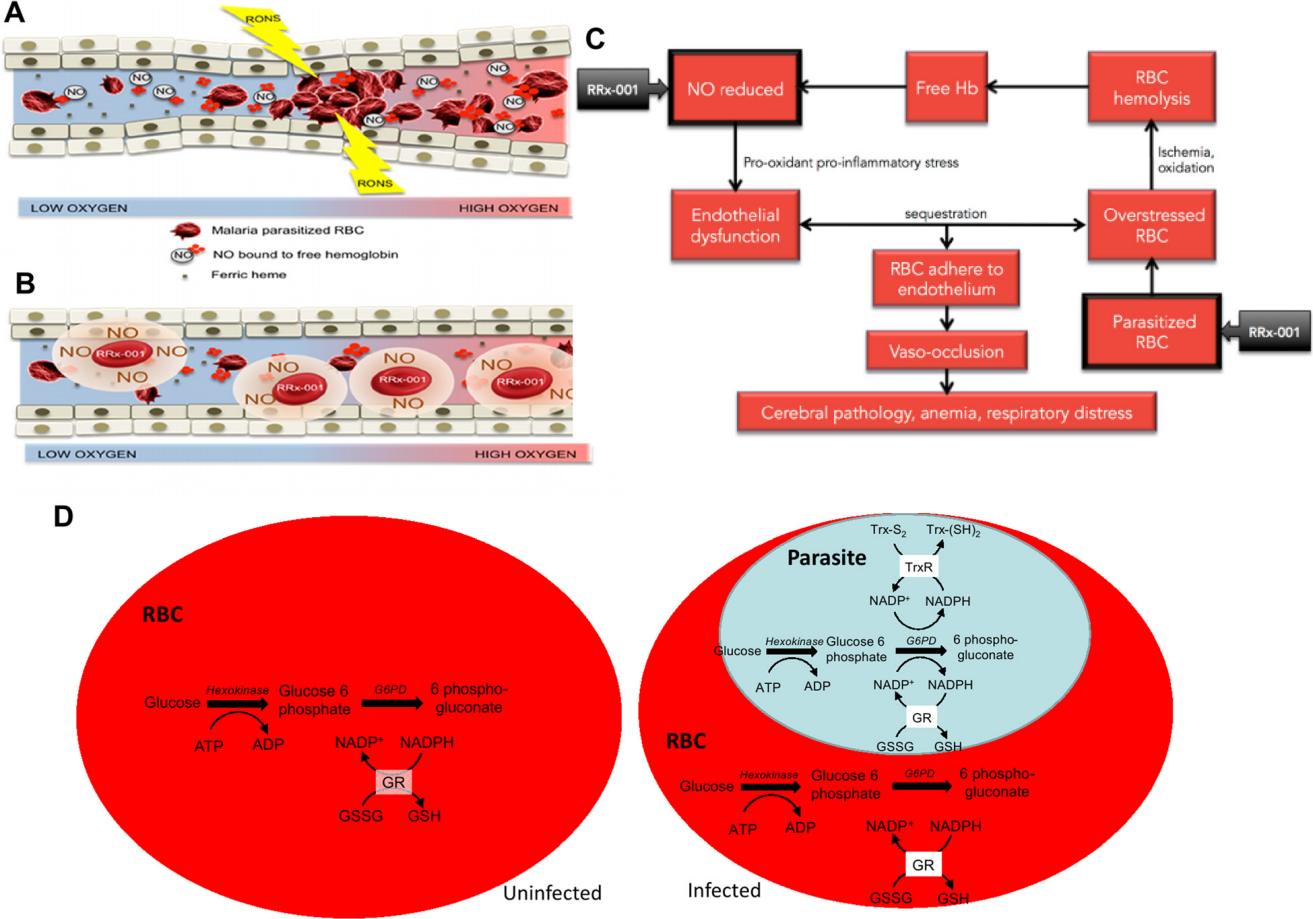
Vascular effects of malaria parasitized red blood cells. **a**. Malaria limits NO bioavailability and RRx-001 restores NO. Malaria is a disease of low NO bioavailability resulting from both low levels of production and high scavenging rates.[45] Red blood cell (RBC) lysis leads to the release of Hb and arginase: Acellular Hb scavenges NO and arginase degrades arginine, which is an NO precursor. The consequences of low NO bioavailability are: less deformable RBCs leading to occlusion, vasospasm and ischemia. As deoxyHb nitrite reductase catalyst, RRx-001 produces NO “on demand” in ischemic/ hypoxic areas, reversing the vascular defects. **b**. Proposed vascular status of malaria parasitized RBCs after treatment with RRx-001. Overview of direct and indirect therapeutic effects of RRx-001 on malarial pathogenesis. RRx-001 affects parasitized RBC directly by increasing of oxidative stress with resulting G6PD inhibition while indirectly treating vasculopathic sequelae due to low NO bioavailiability. **c**. G6PD in uninfected and infected RBCs. In uninfected erythrocytes, G6PD operates at a fraction of its capacity. Under increased oxidative stress, the NADPH is oxidized and G6PD becomes highly activated. Limiting G6PD lowers the ration NADPH/NADP, increasing G6PD activity. Under conditions of oxidative stress, known to be important for the action of anti-malarial drugs, cells with limited G6PD can only activate their G6PD activity to a small extent, under the risk of haemolytic anemia. RBCs and malaria parasites are equipped with antioxidant defense systems, glutathione (GSH) and thioredoxin (Trx) systems, which are both NADPH dependent. The GSH system reduced GSSG to GSH, using GSH reductase (GR), present in RBCs and malaria parasites. In addition, parasites have thioredoxin reductase (TR), which reduces Trx disulfide (Trx-S_2_) to the Trx dithiol-form [Trx-(SH)_2_]. The PPP is the only source of NADPH in erythrocytes, and most likely the major source of NADPH in the parasite

Moreover, treatment with systemic NO donors like nitroglycerin may theoretically lead to cerebral vascular steal syndrome, paradoxically reducing perfusion to ischemic areas and worsening outcome [39], also was not observed with RRx-001 based in human clinical trials. Finally, unlike nitroglycerin, RRx-001 has a specific tropism for RBCs where it binds to Hb and glutathione, initiating a subhaemolytic free radical cascade. These ROS and reactive oxygen nitrogen species (RONS) exacerbate artemether-induced oxidative injury to the parasite at the point of origin in the RBC, tipping them over the edge (Fig. 5) and leads to inhibition of G6PD expression and activity. Replenishment and restoration of NO production “on demand” in hypoxic areas should a) reverse vasospasm and reduce the sequelae of endothelial dysfunction and b) mediate antiplasmodial toxicity through the production of inflammatory levels of ROS and RONS as NO combines with malaria-generated superoxide (O_2_^−^) in the RBC to form highly reactive, peroxynitrite (ONOO^−^) [31, 40]. This reactive free radical, in turn, oxidizes cysteine-dependent enzymes, G6PD and G6PD-6PGL on which the parasite is dependent. An overview of RRx-001 on malarial pathogenesis is shown in Fig. 6.

In treating the ECM only after clear signs of neurological derangement became evident, these studies were designed to closely mimic the typical presentation of a cerebral malarial patient, who is more likely to receive treatment during an advanced disease stage. These data demonstrate that after infection with *P. berghei*, RRx-001 in combination with artemether, reversed vasospasm and improved cerebral perfusion, while significantly decreased parasitaemia and increased survival. These effects were associated with the inhibition and down-regulation of G6PD activity and improved cerebral haemodynamic parameters with significant recovery of motor scores. RRx-001 has anti-malarial activity as a single agent and potent G6PD inhibition at relatively low dose. As an epigenetic agent, which chemosensitizes in cancer, RRx-001 may potentiate the activity of artemether through silencing genes up regulated in responses to stress. Although this hypothesis was not investigated in the current study, future studies will determine the genes methylated by RRx-001 or its byproducts that enhanced the effects of conventional anti-malaria treatment (artemether) and provided microvascular protection.

The pathology of CM is clearly multifactorial involving the interplay of local infection and post-infection systemic complications resulting from aggregation of parasitized RBCs, cytokine release and Hb-driven oxidative damage in the cerebral vasculature. The alleviation of the parasite burden in CM with artemether does not necessarily imply protection against long-term neurological deficits [32], since cerebral ischemia-reperfusion injury following relief of vascular obstruction may lead to coagulopathy, vasoconstriction, and hypoperfusion. Therefore, the key to successfully manage CM is to eliminate or alleviate both the parasitic infection and the vasculopathy, consisting of malarial RBC adhesion and vaso-occlusion. These data suggest that a combination of RRx-001 with artemether would address both the infection and the vasculopathy leading to a promising novel treatment paradigm for CM.

Animal models provide valuable biological information in malaria. Nearly all evaluated interventions have been dramatically effective in the murine model, although there is no convincing evidence of benefit for any adjunctive intervention in human cerebral malaria [7]. Several cases of haemolysis induced by anti-malarial therapies have been reported in severe G6PD deficient patients [6]. Haemolytic potential of RRx-001 in uninfected animals was compared to artemether and the combination RRx-001 and artemether at the dose used with the infected mice. All drugs and their combination did not result in haemolytic crisis (Fig. 6c). In erythrocytes, G6PD only works at a fraction of its maximum capacity, because it is inhibited by NADPH under physiological conditions [8]. Only in severe G6PD-deficient cells, the NADPH/NADP ratio is lower compared to normal cells and the G6PD activity is closer to the maximum activity available [41]. It is unlikely that RRx-001 will produce haemolysis due to G6PD inhibition, because the dose required for treatment only has limited G6PD inhibition.

## Conclusion

In conclusion, in the present study, RRx-001 is an effective adjunctive therapy for ECM in association with artemether; this effect was associated with improvement of vascular dysfunction and motor scores. Evidence of frontline-based artemisinin (qinghaosu) resistance to malaria across Southeast Asia [42] underscores the need for an expanded repertoire of anti-malarials with novel mechanisms of action such as RRx-001. In addition, since epigenetic agents are associated with the reversal of chemoresistance in cancer, it is also possible that epigenetic agents, like RRx-001, may have use in malaria and similarly reverse resistance to the anti-malarials currently in use. While these results are promising, further studies, especially in G6PD-deficient models, to support the potential applicability of RRx-001 as an adjunctive therapy for CM are warranted.
